# Baricitinib induces LDL-C and HDL-C increases in rheumatoid arthritis: a meta-analysis of randomized controlled trials

**DOI:** 10.1186/s12944-019-0994-7

**Published:** 2019-02-18

**Authors:** Chengfeng Qiu, Xiang Zhao, Lang She, Zhihua Shi, Ziwei Deng, Liming Tan, Xiaojun Tu, Shilong Jiang, Bin Tang

**Affiliations:** 1 Departement of Evidence-base Medcine and Clinical center, The First People’s Hospital of Huaihua of University of South China, Huaihua, 418000 People’s Republic of China; 2 Department of Pharmacology, The First People’s Hospital of Huaihua of University of South China, Huaihua, 418000 People’s Republic of China; 3 Department of General Practice, The First People’s Hospital of Huaihua of University of South China, Huaihua, 418000 People’s Republic of China; 4 Department of Pharmacology, The Second People’s Hospital of Huaihua City, Huaihua, 418000 People’s Republic of China; 5 Department of Clinical Pharmaccology, Xiangya Hospital Central South University, Changsha, 410008 People’s Republic of China

**Keywords:** Baricitinib, Rheumatoid arthritis, Low-density lipoprotein cholesterol, High-density lipoprotein cholesterol, Cardiovascular risk

## Abstract

**Background:**

Baricitinib, an oral-administrated selective inhibitor of the JAK1 and JAK2, is recently approved for rheumatoid arthritis (RA) treatment. With the aim to provide some insights on the clinical safety, the current study mainly focused on the effect of baricitinib on low-density lipoprotein cholesterol (LDL-C) and high-density lipoprotein cholesterol (HDL-C) levels and cardiovascular risk.

**Methods:**

The net change scores [least squares mean (LSM) and mean change] of LDL-C and HDL-C levels from baseline with the comparison of baricitinib versus placebo were pooled, respectively. Risk rations (RR) of major cardiovascular events (MACEs) and differences of cardiovascular risk scores at the end of treatment across groups were compared.

**Results:**

Six trials with randomized 3552 patients were finally included in summary analysis. Results showed that baricitinib significantly increased LDL-C levels, the net mean change was 13.15 mg/dl with 95% CI 8.89~17.42 (I^2^ = 0) and the net LSM was 11.94 mg/dl with 95% CI 7.52~16.37 (I^2^ = 84%). HDL-C also increased obviously with the net LSM change was 7.19 mg/dl (95% CI, 6.05~8.33, I^2^ = 47%) and net mean change was 5.40 mg/dl (95% CI, 3.07~7.74, I^2^ = 10%). Subgroup and meta-regression analysis demonstrated baricitinib induced LDL-C and HDL-C increases in a dose-response manner. However, both the pooled RRs of MACEs and differences of cardiovascular risk scores were not statistically significant across groups.

**Conclusion:**

This study confirmed that baricitinib induced a stable dose-response increase in LDL-C and HDL-C levels. Since the causality association between altered lipids and cardiovascular risk was not identified yet, this issue cannot be completely dismissed. Future research is needed to fully dissect the implications of these lipid changes.

**Electronic supplementary material:**

The online version of this article (10.1186/s12944-019-0994-7) contains supplementary material, which is available to authorized users.

## Background

Rheumatoid arthritis (RA) is a chronic, systematic autoimmune disease characterized by synovial inflammation and joint damage [1]. For a long time, conventional synthetic disease-modifying antirheumatic drugs (cDMARDs), usually methotrexate (MTX), ranked as the frontline therapy for RA targeting to improve synovitis and physical function [2]. However, many patients do not achieve the therapy goal, failing to continue therapy because of seriously adverse effect or lose response over time. These issues highlight the need to develop additional therapeutic strategies for RA. Recent advances put emphasis on the crucial role of cytokine network involved in the pathogenesis of RA [2]. Janus kinases (JAKs) are part of the tyrosine kinases family that regulate a variety of signaling cascades of cytokine, many of which are contributed to the RA progression. A new class of small molecules targeting JAKs are successfully developed to be an additional therapy for RA patients [3].

Baricitinib is an oral-administrated selective inhibitor of the JAK1 and JAK2. As the second JAK inhibitor, baricitinib recently attracted numerous attention for the approval of RA treatment in European Union, Japan and USA [4]. Completed phase II and III trials demonstrated baricitinib significantly improves symptoms of moderate-to-severe active RA patients who had undergone an inefficient response or intolerance to cDMARDs, presenting a therapy value of baricitinib in a new field of RA treatment [5].

RA is strongly associated with high risk of cardiovascular (CV) events. A pooled analysis demonstrated the CV risk was 48% higher in patients with RA than those in general individuals [6]. The mechanism underlying the excess risk of CV events in RA remains unclear, but the traditional risk factors, systematic inflammation and the other RA-specific (mainly the disease activity) may play a role in this increased risk [7]. Particularly, recognition of CV risk of anti-rheumatism treatment was important to guide the clinical management of RA. Both MTX and tumor necrosis factor (TNF) inhibitor (infliximab) are associated with decreased CV risk [8, 9]. The cardiovascular risk associated with non-steroidal anti-inflammatory drugs (NSAIDs) was modest lower than in non-RA individuals [10]. As a new class of agent for RA, the safety of baricitinib, especially their effects on cardiovascular risk and associated risk factors, remains to be assessed. For now, the follow-up duration of completed phase II and III trials were 12~52 weeks. Such short follow-up duration is inappropriate for assessment of CV risk. A long-term observation is deserving to be held and the associated risk should be estimated.

Dyslipidemia, especially the elevated low-density lipoprotein cholesterol (LDL-C) levels which is regarded as “bad cholesterol”, has been well established as the major risk of CV disease [11, 12]. In contrast, serum high-density lipoprotein cholesterol (HDL-C) is considered to be protective against cardiovascular disease with the name of “good cholesterol” [11, 12]. With the aim to provide some insights on the clinical safety of baricitinib, the current study mainly focuses on the effect of baricitinib with different dose on LDL-C and HDL-C levels and potential cardiovascular risk.

## Materials and methods

We followed the guidelines proposed by the Cochrane handbook for performing and reporting the current meta-analysis [13].

### Search methods and resources

Studies that reported the effect of baricitinib on plasma lipids in patients with rheumatoid arthritis were considered as our interest. Two investigators independently searched the relevant studies across these databases: Medline, Embase, and the Cochrane Central Register of Controlled Trials (CENTRAL) and websites (www.clinicaltrials.gov). The following key words were used for searching: (JAK inhibitor OR baricitinib OR LY3009104 OR INCB028050) AND (“rheumatoid arthritis” OR RA). The details of search algorithm of Medline (Via PubMed) was provided in Additional file 1. In addition, we also extend the search by scrutinizing the reference lists from all relevant studies or reviews. The last study search was updated in 1 October 2018.

### Study selection and data extraction

Two investigators (She L and Jiang SL) independently identified eligible trials by carefully scrutinized titles, abstracts and full text. Consensus was achieved through consulted with the third investigator in case of disagreement. Original studies were considered as eligible if they meet the pre-defined inclusion criteria: (i) patients were 18 years of age or older and had active rheumatoid arthritis; (ii) the comparation were the baricitinib versus placebo or active control with or without any background therapy; (iii) reported outcomes include the change scores of LDL-C and HDL-C, the estimation of potential cardiovascular risk at the end of treatment; (iv) clinical trials of randomized, double-blind, placebo- or active-controlled design. Of note, any studies that with duplicated reporting, important information missing or failed to meet any of the inclusion criteria listed above were excluded.

Two investigators (She L and Jiang SL) extracted the following data independently from the primary text of individual trial: trial name, recruitment period, number of centers, publication year, patients’ demographics and clinical characteristics, follow-up duration, duration of RA, randomized patients, background therapy, the change values of LDL-C and HDL-C at the end of treatment from baseline.

### Outcomes

In this study, we focused on the net change of LDL-C and HDL-C levels at the end of follow-up over baricitinib treatment when compared to the placebo or the active-control. Net change scores were expressed as the net least squares mean (LSM) and net mean change at the end of follow-up from baseline, the two scores were pooled separately in the current meta-analysis. Net mean change scores were calculated as: (measure at end of follow-up in the treatment group−measure at baseline in the treatment group) − (measure at end of follow-up in the control group−measure at baseline in the control group). LSM means adjusted mean in primary text, and is apparently more reasonable because the potential confounders have been adjusted. In additional, with the aim to assess the potential cardiovascular risk of baricitinib, we calculated the risk of MCVEs and the differences of CV scores with the comparations of baricitinib versus placebo or versus active agents. Formulas used for data transformation were listed in Additional file 2.

### Bias risk assessment

Two investigators (Deng ZW and Shi ZH) judged the bias risk and quality of included trials by using Cochrane risk of bias assessment tool independently. According to the guidelines, five aspects were assessed: allocation sequence generation, allocation concealment, blinding of participants and investigators, completeness of outcome data, and selective outcome reporting.

### Statistical analysis

In this meta-analysis, we compared the net changes of serum LDL-C and HDL-C levels at the end of treatment from baseline in baricitinib group to those in control group. Net LSM change and net mean change of LDL-C and HDL-C levels were pooled using the DerSimonian-Laird random-effects model, respectively. For studies containing multiple intervention with different dose, we combined them to create a single pairwise comparison by using a weighted average [13]. Pooled effect sizes were represented as weight difference (WMD) and 95% confidence interval (CI). To estimate the CV risk, the CV risk scores (Framingham risk score and Reynolds risk score) of patients with different treatment were compared and numbers of MACEs in each group were extracted to calculate risk rations (RRs). In order to assess the robustness of pooled results and identify the potential contributor of heterogeneity, sensitivity analysis was conducted using the leave-one-out method in each turn to investigate the influence of single study on the overall risk estimate. In view of dose-dependent and time-dependent clinical effect with most of drugs, we performed a subgroup analysis and a random-effects meta-regression to evaluate the association between baricitinib-induced elevation of plasma LDL-C and HDL-C levels with treatment dose and duration.

Between-study heterogeneity was quantitatively assessed using the I^2^ index [14], values of 25, 50, and 75% presented as mild, moderate, and high heterogeneity. Potential publication bias was explored by visual inspection of funnel plots for asymmetry [15] and statistical evaluation with Begg’s rank correlation test [16] and Egger’s linear regression test [17]. Two-tailed α level of significance was set at 0.05.

All statistical analyses were performed with Review Manager Version 5.3 (The Nordic Cochrane Center, Copenhagen, Denmark) and STATA/SE.12.0 (StataCorp, College station, Texas, USA).

## Results

### Baseline characteristics of included trials

**Fig. 1:**
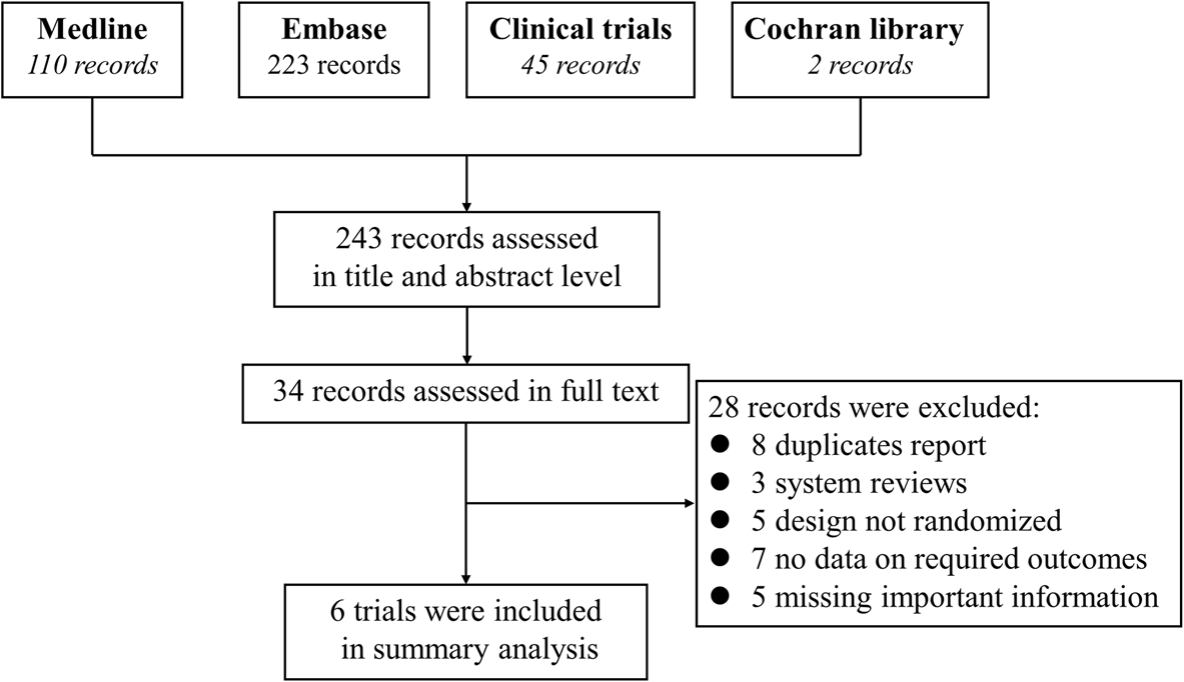
Flow diagram of study selection process

After a critically reviewed the title, abstract and full text according to the predefined criteria, six trials were finally included in the current meta-analysis, including four III phases trials (BRA-BUILD, RA-BEGIN, RA-BEACON and RA-BEAM) and two II phases trials (NCT01469013 and NCT01185353). Flow diagram of study selection process is presented in Fig. 1. Except one trial performed as single center study [18], all the rest are multicenter studies. A total of 3552 RA patients were randomized into 5 arms: control, and baricitinib with different dose (1 mg, 2 mg, 4 mg and 8 mg). Among them, 78% of patients were female (*n* = 2788). Except one trial that included the patients with a short RA duration (ranged from 1.3~1.9 years) and receiving no prior DMARDs therapy, the rest patients had a long RA duration (duration of RA ≥5 years) and has undergone an insufficient response or intolerance to ≥1 DMARDs. Most patients (*n* = 2964) have added a stable background therapy including any DMARDs in addition to the placebo or baricitinib, there are 588 patients who received no background therapy. Follow-up duration ranged from 12~52 weeks. More details of study characteristics were presented in the Table 1, and the characteristics of included studies for analysis of other JAK inhibitors were displayed in Additional file 3.

**Table 1:** Study characteristics of included trials

Trial name	Publication year	Recruitment period	No. of centers	Patients characteristic	Follow-up (w)	Randomized patients	Age (Year)^a^	Female n(%)	Duration of RA (year)	Background therapy	Control	Baricitinib
RA-BEAM [28]	2017	2012.11–2014.09	281	Active RA patients had an inadequate response to methotrexate	24~52	1307	53 .4 (6.3)	1008 (71)	10	MTX	Placebo	4 mg, qd
RA-BEGIN [29]	2017	2013.01–2014.08	198	RA patients who received no prior DMARDs therapy	24~52	588	50.2 (13.4)	425 (72)	1.3~1.9	Non	Placebo	4 mg, qd
BRA-BUILD [30]	2016	2013.01–2014.05	182	RA patients who had insufficient response or intolerance to ≥1 DMARDs	12~24	684	51 (12)	557 (81)	8	Any DMARD	Placebo	2 mg and 4 mg, qd
RA-BEACON [31]	2016	2013.01–2014.03	178	RA patients who had insufficient response or intolerance to ≥1 DMARDs	12~24	527	56 (11)	431 (82)	14	Any DMARD	Placebo	2 mg and 4 mg, qd
NCT01469013 [32]	2016	2011.11–2013.12	24	RA patients with stably using of MTX	12	145	53.7 (12)	118 (81)	5~6.3	MTX	Placebo	1 mg, 2 mg, 4 mg and 8 mg, qd
NCT01185353 [33]	2015	2010.10–2012.02	69	RA patients with regularly using of MTX	12	301	53.3 (11.7)	249 (83)	5.3~6.6	Any DMARD	Placebo	1 mg, 2 mg, 4 mg and 8 mg, qd

^a^Mean (SD); Notes: RA, rheumatoid arthritis; MTX, methotrexate; DMARD, disease-modifying antirheumatic drugs

### Baricitinib induces LDL-C and HDL-C increases in patients with RA

**Fig. 2:**
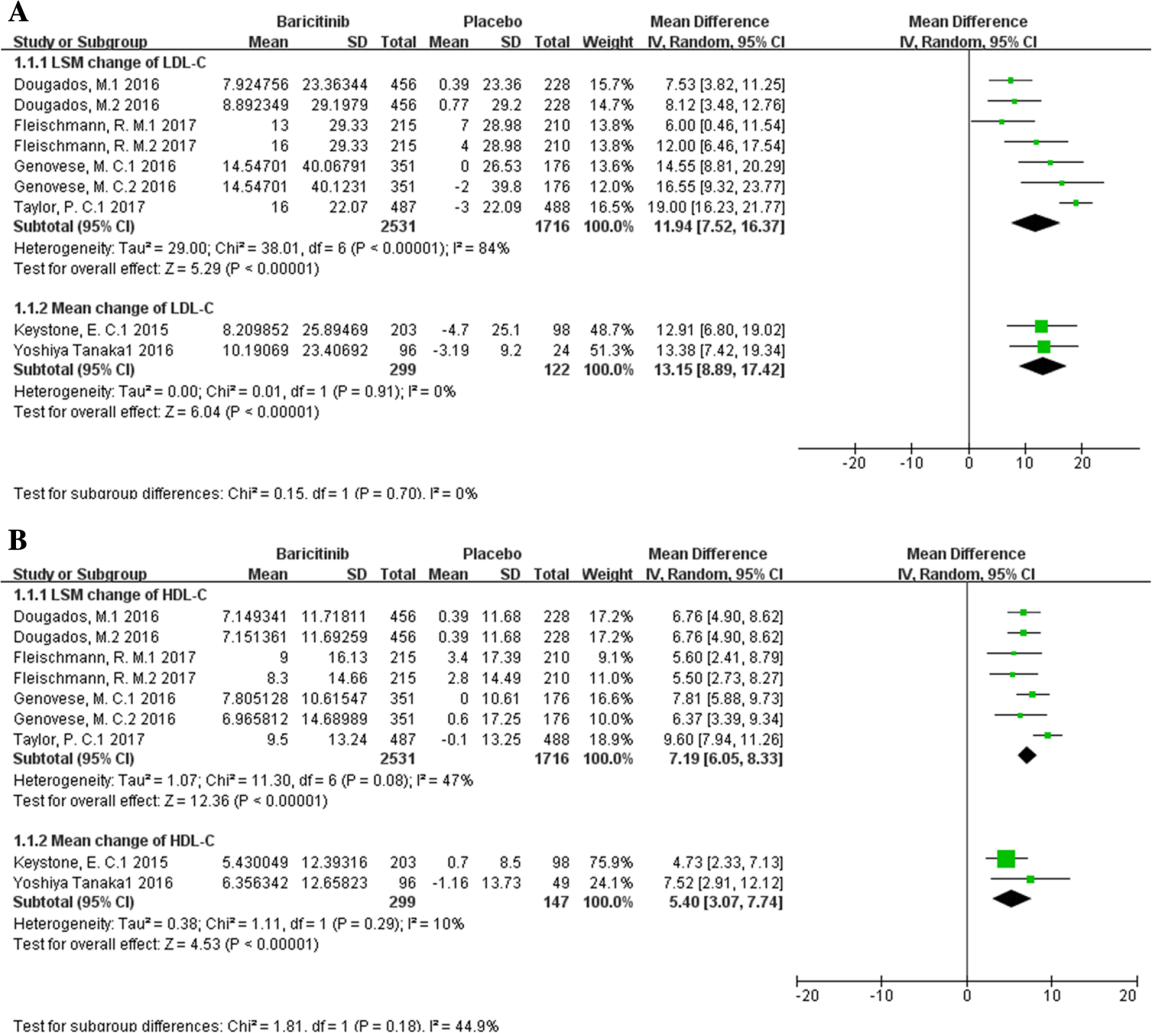
Funnel plot of the effect of baricitinib on LDL-C levels and HDL-C levels.Net change scores of LDL-C and HDL-C levels were pooled with the comparation of baricitinib versus placebo. **a** Pooled estimates of net change score of LDL-C level; **b** Pooled estimates of net change score of HDL-C level. Notes, ES, effect size; LSM, least squares mean

To systematically and completely evaluate the effect of baricitinib on LDL-C, we compared net change scores of LDL-C levels in patients with baricitinib treatment to this in patients with placebo (Fig. 2a). Pooled results showed that baricitinib significantly increased the levels of serum LDL-C after treatment for 6~52 weeks regardless of the previous treatment (DMARDs-naïve or resistant) when compared with placebo, the net mean change was 13.15 mg/dl (95% CI, 8.89~17.42, I^2^ = 0), even adjusted the potential confounders, the net change scores which presented as net LSM remained significantly (net LSM, 11.94 mg/dl, 95% CI, 7.52~16.37, I^2^ = 84%).Similar to LDL-C, HDL-C increased obviously in RA patients seen after baricitinib treatment (Fig. 2b), with the net LSM change was 7.19 mg/dl (95% CI, 6.05~8.33, I^2^ = 47%) and net mean change was 5.40 mg/dl (95% CI, 3.07~7.74, I^2^ = 10%). Sensitivity meta-analysis showed these pooled results of net LSM change of LDL-C (Additional file 4: A) and HDL-C (Additional file 4: B) were robust with no significant change by any single study, but the heterogeneities have greatly reduced after excluded one study, while the I^2^ reduced to 52% from 84% in the estimation of LDL-C change and the I^2^ reduced to 0 from 47% (Additional file 5).

In additional, we also estimated the effect of the other JAK inhibitors on LDL-C (Additional file 6) and HDL-C levels (Additional file 7) in patients with RA. Summary data showed that tofacitinib (pan-JAK inhibitor) and decernotinib (selective JAK3 inhibitor) had much stronger effect on LDL-C increase with the net mean change of 16.84 mg/dl (95% CI, 10.31~23.36) and 15.44 mg/dl (95% CI, 6.58~24.31), respectively. Filgotinib (selective JAK1 inhibitor) moderately increased LDL-C level with the net mean change of 9.59 mg/dl (95% CI, 3.61~15.57). Surprisingly, results demonstrated peficitinib (mainly moderately inhibits JAK3) did not have obvious effect on LDL-C levels (net mean change, 0.77 mg/dl, 95% CI, − 0.47~2.01). As for the assessment of HDL-C levels, the net mean changes for tofacitinib, decernotinib and filgotinib were 8.09 mg/dl (95% CI, 5.40~10.79), 3.25 mg/dl (95% CI, 1.00~7.51) and 4.24 mg/dl (95% CI, 0.69~7.78), respectively. Of note, peficitinib significantly increased HDL-C levels (net mean change, 6.64 mg/dl, 95% CI, 0.72~12.57), other than its effect on LDL-C.

### Baricitinib-induced increases of LDL-C and HDL-C are mainly associated with treatment dose

**Fig. 3:**
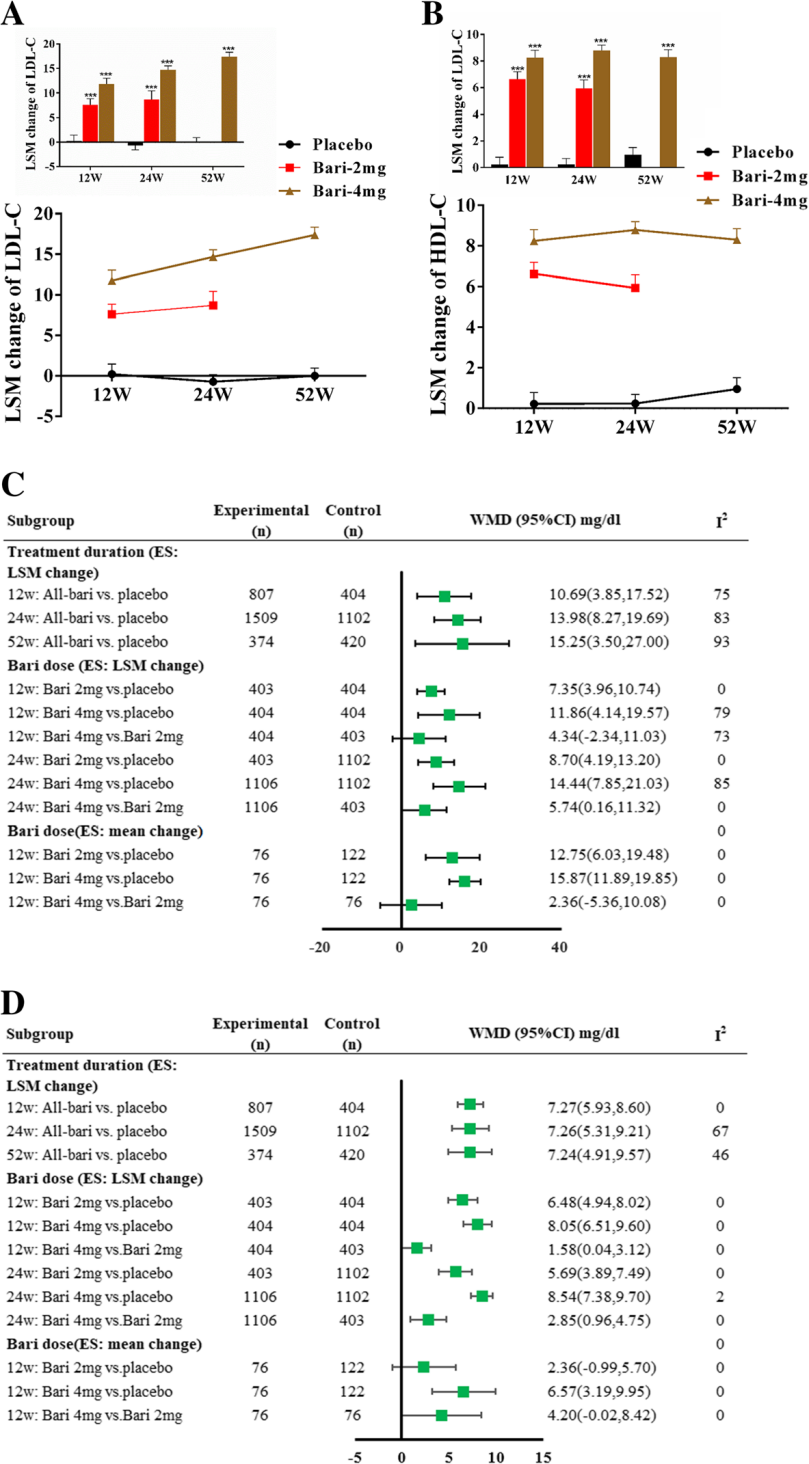
Subgroup analyses sorted by treatment dose and duration. **a** and **b** LSM changes of LDL-C and HDL-C levels across placebo, baricitinib 2 mg and 4 mg from week 12 to week 24 to baseline. Values presented as LSM ± SE. vs. placebo, *** *p* < 0.001; **c** Subgroup analysis of LDL-C changescores; **d** Subgroup analysis of HDL-C change scores. Notes, Bari, baricitinib; WMD, weighted mean difference; LSM, least squares mean. SE, standard error

Baricitinib was recently approved for RA treatment. However, baricitinib 4 mg was not permitted in USA now. In this section, we mainly focused on the potential dose- and time -dependent response on LDL-C and HDL-C levels. Figure 3a and b presented the LSM change of LDL-C and HDL-C for placebo, baricinitib 2 mg and 4 mg at the week 12, 24, 52, respectively. It seemed that the increased LDL-C and HDL-C levels which induced by baricitinib were more related to treatment dose than that related to treatment duration. We further performed a subgroup analysis of LDL-C change (Fig. 3c) and HDL-C change (Fig. 3d) stratified by treatment duration and treatment dose. We observed that the net LSM changes of LDL-C for baricinitib 2 mg and 4 mg compared to placebo was increased as the treatment duration was extended (12-week to 52-week), however, the net LSM change of HDL-C remained stable across baricinitib 2 mg and 4 mg from week 12 to week 52.

When compared to placebo, the net change scores of LDL-C (Fig. 3c) and HDL-C (Fig. 3d) for baricinitib 4 mg was much higher than that for baricinitib 2 mg not only at week 12 but also at week 24. We further compared baricitinib 4 mg with 2 mg, net LSM change of LDL-C was 4.34 mg/dl (95% CI, − 2.34~11.03) and 5.74 mg/dl (95% CI, 0.16~11.32) at week 12 and week 24, and the net mean change of LDL-C was 2.36 mg/dl (95% CI, − 5.36~10.08) at week 12; net LSM change of HDL-C was 1.58 mg/dl (95% CI,0.04~3.12) and 2.85 mg/dl (95% CI,0.96~4.75) at week 12 and week 24, and the net mean change of HDL-C was 4.20 mg/dl (95% CI,-0.02~8.42) at week 12.

**Fig. 4:**
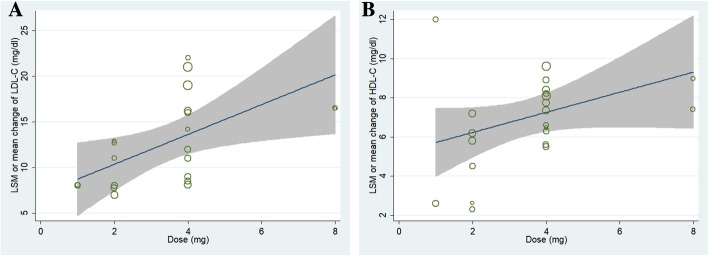
Meta-regression of the association between baricitinib dose and elevated LDL-C (**a**) and HDL-C levels (**b**)

Random–effects meta-regression suggested a significant association between baricitinib-induced elevation of LDL-C and HDL-C level with treatment dose [LDL-C (Fig. 4a): adjusted R^2^ = 23.6%, slope = 7.01, 95% CI, 1.64~12.38; HDL-C (Fig. 4b): adjusted R^2^ = 34.64%, slope = 5.17, 95% CI, 2.92~7.43)]. But no significant association was found between LDL-C and HDL-C elevation with duration of treatment (data not show).

### Cardiovascular risk assessment of baricitinib treatment in patients with RA

**Fig. 5:**
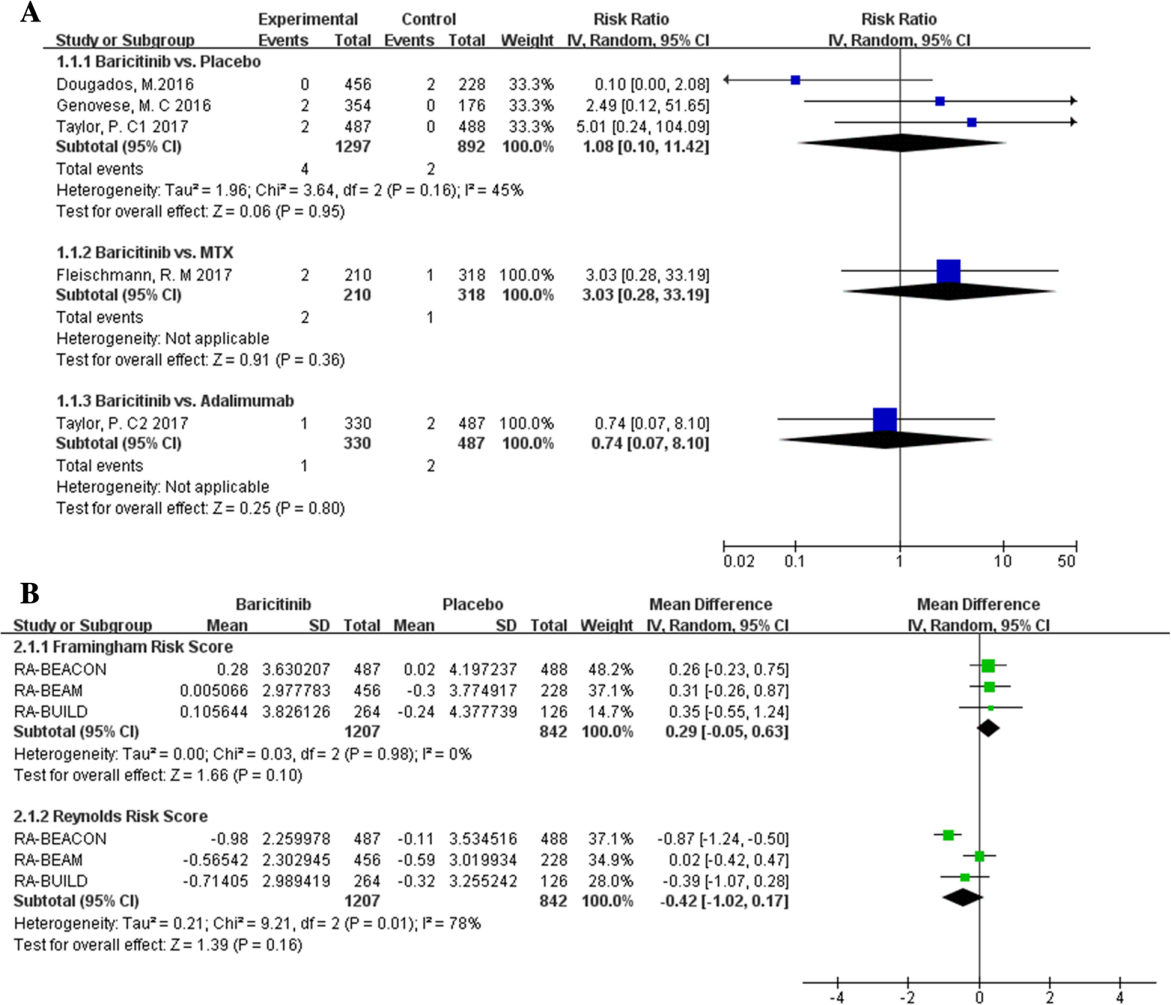
Pooled estimates of the relative risk of the incidence of major cardiovascular events (**a**) and the differences of cardiovascular risk scores (**b**)

Aimed to assess the CV risk of baricitinib treatment, first, we calculated the risk of MACE in patients with baricitinib treatment compared to placebo or the other active agents (MTX and adalimumab). As showed in Fig. 5a, pooled RR was 1.08 with 95% CI 0.10 to 11.42 (baricitinib vs. placebo), 3.03 with 95% CI 0.28 to 33.19 (baricitinib vs. MTX) and 0.74 with 95% CI 0.07 to 8.10 (baricitinib vs. adalimumab), but no statistically significant was found across all comparations. Furthermore, we observed no significant differences of CV risk scores at week 12 (both for Framingham risk score and Reynolds risk score) between the baricitinib and placebo group (Fig. 5b).

### Bias assessment

**Fig. 6:**
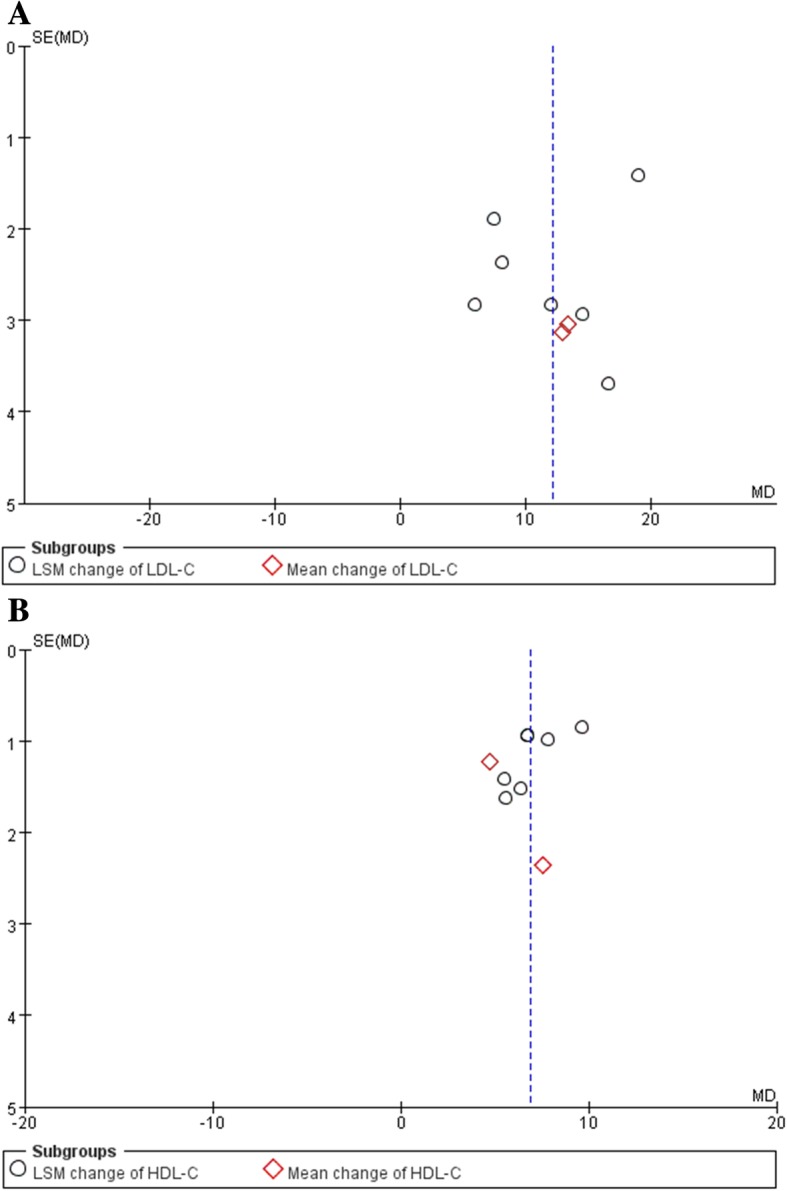
Funnel plot of baricitinib and LDL-C level (**a**) and HDL-C level (**b**)

Overall risk of bias was ranked as low in included trials (Additional file 8). There was no obvious publication bias was assessed by visual inspection of funnel plots for asymmetry (Fig. 6) and through Begg’s rank correlation test (*p* values for LDL-C and HDL-C estimation were 0.36, 0.17, respectively) and Egger’s linear regression test (p values for LDL-C and HDL-C estimation were 0.34, 0.07, respectively).

## Discussion

The salient findings of this meta-analysis of 6 randomized controlled trials (RCTs) including 3552 randomized patients with RA can be listed as follows. First, baricitinib treatment regardless of 2 mg and 4 mg significantly induces LDL-C and HDL-C increases in patients with RA when compared with placebo both at week 12, 24 and 52. Second, baricitinib-induced increased in LDL-C and HDL-C were strongly associated with the treatment dose but not with the treatment duration, suggesting a dose - response manner of baricitinib in inducing LDL-C and HDL-C increases. Third, there was no significant differences of CV risk between baricitinib and placebo groups during the follow-up of 52 weeks.

Patients with RA are strongly associated with increased risk of CV disease which could hardly be fully explained by traditional risk factors [6]. Further adding to the confusion, active RA present a fall in both LDL-C and HDL-C levels which called “lipid paradox”- decreased lipids and increased CV risk [19]. Systemic inflammation is proposed to play a role in the increased CV risk and also in the altered lipid metabolism associated with RA [19]. Anti-inflammatory therapy with TNFα-inhibitor (adalimumab) induced an elevation of LDL-C and HDL-C mildly seen after treatment, confirming the potential role of inflammation in lipid metabolism [20]. Results of the current study also showed that various JAK inhibitors except to peficitinib all lead to an elevation both of LDL-C and HDL-C levels. Of note, these increases induced by JAK inhibitors were much higher than those induced by adalimumab, studies also demonstrated that adalimumab-induced lipids level is transient [21] while JAK inhibitors-induced LDL-C and HDL-C elevation lasted for the full period of treatment, these results suggesting that suppression of inflammation just partially underlies the increases in lipids levels, factors specific to different treatment may have a strong influence on the degree and pattern of lipid profile change. Even across the various JAK inhibitors, the change levels of LDL-C and HDL-C were much different from each other. Among them, tofacitinib (a pan-JAK inhibitor), the first JAK inhibitor, had the strongest increases both in LDL-C and HDL-C levels. Baricinitib (inhibitor of JAK1 and JAK2), newly licenced for the treatment of RA, also lead to significant elevation of LDL-C and HDL-C. Surprisingly, peficitinib, moderate selectivity for JAK-3 inhibition, obviously increased HDL-C level but had no change in LDL-C levels, providing some additional insight for understanding the role of different JAK signaling in cholesterol metabolism. In additional, the activation of JAK signaling are demonstrated that involved in the pathological process of many cancers [22, 23]. Epidemiological studies showed that lipids level including total cholesterol (TC), LDL-C and HDL-C all much lower in these patients with cancer than those in general population [24]. Though these sighs could not confirm the direct link between JAK activation and lower levels of lipids, this information have raised our awareness regarding whether JAK signaling pathway involved in cholesterol metabolism.

Baricinitib, a selectively inhibitor for JAK1 and JAK2, has been approved for RA treatment in European Union and Japan in 2017 [4]. Recently, considering the insufficient clinical safety data, just baricinitib 2 mg but not 4 mg was approved for RA treatment in USA. In the current study, we explored the dose-response of baricinitib on lipids changes, both subgroup studies stratified by dose and meta-regression all demonstrated consistent results that baricinitib lead to a stable dose-dependent increases in LDL-C and HDL-C levels. Particularly, baricinitib-induced LDL-C change gradually increased as the extended duration of treatment (week 12 to 52), however, the HDL-C increase was similar at the point of week 12, 24 and 52, suggesting the LDL-C elevation was associated with baricinitib dose and treatment duration but HD-C elevation was mainly associated with dose.

RA is an independent risk factor for CV disease [25]. However, the following are some questions needing evaluation in the changed lipids and CV risk field: (i) whether the increased LDL-C level further increased the risk of CV disease in patients with RA; and (ii) whether the increased HDL-C perform a protective role against CV disease. Particularly, it should also be mentioned that CV risk is not only dependent on a particular cholesterol level, but also is strongly depend on the composition of lipoporteins [26], which is not routinely analyzed in daily clinical practice. Here we compared the risk of MACE at the end of treatment, though there was no significant differences across the baricitinib, MTX and adalimumab, the result should be interpreted with caution because of short follow-up duration, which is limited to observe the incidence of CV events. Furthermore, we calculated the net change of Framingham and Reynolds CV risk scores at week 12 to estimate the CV risk. Pooled results also displayed no significant difference with the comparation of baricitinib versus placebo. Actually, CV risk is underestimated in the patients with RA using these calculators [27], indicating that the CV risk estimation based on the current available studies could hardly reflect the reality. In this respect, long-term observational data will be important across the field.

Some limitations of the current meta-analysis should be recognized. First, though the stable of dose-response of baricitinib on LDL-C and HDL-C levels was confirmed, the underlying mechanism remains further study. Second, our study was not powered to identify the relationship between baricitinib-induced lipid changes and CV risk. Third, the estimation of CV risk of baricitinib remains to be further confirmed with long-term observation.

## Conclusions

This study confirmed that baricitinib induced a stable dose-response increase in LDL-C and HDL-C levels. Since the causality association between altered lipids and CV risk was not identified, however, this issue cannot be completely dismissed. Further research is needed to fully dissect the consequences of these lipid changes and how baricitinib modulate cholesterol metabolism.

## Additional files

Additional file 1: Formulas used in the current study. (DOCX 16 kb)

Additional file 2: Search algorithm from Medline. (DOCX 16 kb)

Additional file 3: The Study characteristics of included trials about JAK inhibitors. (DOCX 42 kb)

Additional file 4: Results of sensitivity analysis. (TIF 361 kb)

Additional file 5: Sensitivity analysis of the effect of baricitinib on LDL-C levels (A) and HDL-C levels (B). (DOCX 25 kb)

Additional file 6: Pooled estimates of net change scores of LDL-C with the comparation of JAK inhibitors versus placebo. (TIF 261 kb)

Additional file 7: Pooled estimates of net change scores of HDL-C with the comparation of JAK inhibitors versus placebo. (TIF 240 kb)

Additional file 8: Risk of bias in the included trials as assessed by the Cochrane risk of bias assessment tool. (DOCX 14 kb)

## Abbreviations

CI: Confidence interval
CV: Cardiovascular
DMARD: Disease-modifying antirheumatic drugs
HDL-C: High-density lipoprotein cholesterol
JA: Janus kinases
LDL-C: Low-density lipoprotein cholesterol
LSM: Least squares mean
MACE: Major cardiovascular events
MTX: Methotrexate
RA: Rheumatoid arthritis
RCT: Randomized controlled trial
RR: Risk rations
TC: Total cholesterol
TNF: Tumor necrosis factor
WMD: Weighted mean difference
