# Supplementary material for: Baricitinib induces LDL-C and HDL-C increases in rheumatoid arthritis: a meta-analysis of randomized controlled trials
Source: Lipids Health Dis. 2019 Feb 18;18:54. doi: 10.1186/s12944-019-0994-7 (PMC6380020; doi:10.1186/s12944-019-0994-7)
Supplement: Supplementary file 1 — Formulas used in the current study. (DOCX 16 kb) [file 12944_2019_994_MOESM1_ESM.docx]

**Additional file 1.** Search algorithm of Medline

| PubMed (Medline) | | |
| --- | --- | --- |
| #1 | JAK inhibitors OR JAK inhibitor OR baricitinib OR LY3009104 OR INCB028050 | 6643 |
| #2 | "rheumatoid arthritis "OR RA | 132930 |
| #3 | #1 AND #2 | 488 |
| #4 | randomized controlled trial[Publication Type] | 471409 |
| #5 | controlled clinical trial[Publication Type] | 559306 |
| #6 | randomized [tiab] | 459226 |
| #7 | randomly [tiab] | 299781 |
| #8 | trial [tiab] | 523583 |
| #9 | #4 OR #5 OR #6 OR #7 OR #8 | 1194503 |
| #10 | (animals [mh] NOT humans [mh]) | 4511745 |
| #11 | #3 AND #9 | 116 |
| #12 | #11 NOT #10 | 116 |
