# Supplementary material for: Baricitinib induces LDL-C and HDL-C increases in rheumatoid arthritis: a meta-analysis of randomized controlled trials
Source: Lipids Health Dis. 2019 Feb 18;18:54. doi: 10.1186/s12944-019-0994-7 (PMC6380020; doi:10.1186/s12944-019-0994-7)
Supplement: Supplementary file 2 — Search algorithm from Medline. (DOCX 16 kb) [file 12944_2019_994_MOESM2_ESM.docx]

**Formulas used in the current study**

In the current study, the formulas used for data transformation were listed as follows [1]:

1. Net change scores = (measure at end of follow - up in the treatment group − measure at baseline in the treatment group) − (measure at end of follow - up in the control group − measure at baseline in the control group).
2. The estimation of standard error (s.e.) values using the formula: s.e.=$s.d.\div\surd N$.

N means the number of samples. s.d. means the standard deviation.

1. The s.d. value of mean difference were calculated using the following formula:

s.d =$\sqrt{{[({s.d.}_{pre-treatment})}^{2}+{({s.d.}_{post-treatment})}^{2}-2\times R\times({s.d.}_{pre-treatment}\times{s.d.}_{post-treatment})}]$.

R value was assumed as 0.5.

1. If the values were reported as median (m) and range (a, b), formula used for the calculation of mean as follow[3]: mean= $(a+2m+b)\div4$, S2=$\frac{1}{12}\times\{{\frac{\left( a-2\times m+b \right)^{2}}{4}+\left（ b-a \right）}^{2}\}$

Reference

1. Higgins JJ GS. Cochrane Handbook for Systematic Reviews of Interventions. Version 5.1.0 [updated March 2011]. The Cochrane Collaboration, 2011 Available from wwwcochrane-handbookorg 2011.
