# Supplementary material for: Baricitinib induces LDL-C and HDL-C increases in rheumatoid arthritis: a meta-analysis of randomized controlled trials
Source: Lipids Health Dis. 2019 Feb 18;18:54. doi: 10.1186/s12944-019-0994-7 (PMC6380020; doi:10.1186/s12944-019-0994-7)
Supplement: Supplementary file 3 — The Study characteristics of included trials about JAK inhibitors. (DOCX 42 kb) [file 12944_2019_994_MOESM3_ESM.docx]

**Table 1.** The Study characteristics of included trials about JAK inhibitors.

| Trial name | Publication year | Recruitment period | No. of centers | Patients characteristic | Follow-up (week) | Randomized patients | Female n（%） | Age  (Year) | Duration of RA  (year) | Background therapy | Control | JAK inhibitors |
| --- | --- | --- | --- | --- | --- | --- | --- | --- | --- | --- | --- | --- |
| NCT00847613  (III) [1] | 2013 | 2009.03-2011.04 | 111 | Active RA | 24 | 797 | 679  (85) | 52.2  (11.9) ^a^ | 7.5~11.8 | MTX | Placebo | Tofacitinib, 1,3,5,10,15 and 20 mg |
| NCT00960440 (III) [2] | 2013 | NA | 82 | Patients with RA | 12~24 | 399 | 335  (84) | 54-55^b^ | 11.3-13.0 | MTX | Placebo | Tofacitinib  5mg and 10mg, bid |
| NCT00814307  (III) [3] | 2012 | 2009.02-2010.06 | 94 | Patients with RA | 12 | 611 | 528  (86) | 49-52 ^b^ | 7.7-8.6 | MTX | Placebo | Tofacitinib  5mg and 10mg, bid |
| NCT00550446  (IIb) [4] | 2012 | NA | 63 | Diagnosis of RA for ≥6 months | 24 | 384 | 333  (87) | 52-55 ^b^ | 7.7-10.8 | MTX | Placebo | Tofacitinib  1mg, 3mg, 5mg,10mgand 15mg, bid |
| NCT00147498  (IIa) [5] | 2009 | NA | 60 | Patients with RA | 6 | 264 | 226  (86) | 50.6  (11.8) a | 8.7-10.2 | Any Antirheumatic treatment | Placebo | Tofacitinib  5mg,15mgand 30mg, bid |
| NCT00413660  (IIb) [6] | 2012 | NA | 72 | Diagnosis of RA for 6 months | 24 | 507 | 406  (81) | 45-64 ^b^ | 7.5-11.8 | MTX | Placebo | Tofacitinib  1mg, 3mg, 5mg,10mg, 15mg and 20mg bid |
| NCT00853385  (III) [7] | 2012 | 2009.01.30-2011.02.10 | 115 | Active RA | 48 | 717 | 586  (82) | 53.0 (12.1) a | 6.9-9.0 | MTX | Placebo | Tofacitinib  5mg and 10mg, bid |
| NCT01052194  (IIa) [8] | 2015 | 2010.02-2011.07 | 54 | Patients with RA | 12 | 206 | 166  (81) | 56.1  (9.9) a | 7.73 | Any DMARDs | Placebo | Decernotinib  25mg, 50mg,100mg and 150mg, bid |
| NCT2011-004419-22  (IIb) [9] | 2016 | 2012.05- | 103 sites | Patients with RA | 24 | 358 | 289  (81) | 53^c^ | 7.3 | MTX | Placebo | Decernotinib  100mg, 150mg, 200mg,qd and 100mg bid |
| NCT01894516 (IIb) [10] | 2016 | 2013.10-2015.05 | 59 | Diagnosis of RA for ≥6 months | 12-24 | 283 | 231  (82) | 52.3  (12.2) a | 9 | No | Placebo | Filgotinib  50mg, 100mg and 200mg, qd |
| NCT01565655  (IIb) [11] | 2017 | NA | 41 sites | Patients with RA | 12 | 289 | 237(82) | 54-11.3 ^b^ | 10 | csDMARDs | Placebo | Peficitinib  25mg, 50mg, 100mg and 150mg, qd |
| NCT01649999  (IIb) [12] | 2016 | 2012.02-2013.07 | 43 sites | Patients with RA | 12 | 281 | 228(81) | 53  (11.6)a | 7 | Any Antirheumatic treatment | Placebo | Peficitinib  25mg, 50mg, 100mg and 150mg, qd |
| NCT01554696  (IIb) [13] | 2017 |  | 43 sites | Moderate-to-severe RA with an inadequate response to MTX | 12 | 378 | 315(83) | 52-55 ^b^ | 7.2-8.1 | MTX | Placebo | Peficitinib  25mg, 50mg, 100mg and 150mg, qd |

Notes^: a^ presented as mean ± s.d., ^b^ presented as range, and ^c^ presented as mean.

NA, no assessment; RA, rheumatoid arthritis; MTX, methotrexate; DMARD, disease-modifying antirheumatic drugs.

Reference

1. van der Heijde D, Tanaka Y, Fleischmann R, et al. Tofacitinib (CP-690,550) in patients with rheumatoid arthritis receiving methotrexate: twelve-month data from a twenty-four-month phase III randomized radiographic study. Arthritis Rheum. 2013, 65:559-570.

2. Burmester GR, Blanco R, Charles-Schoeman C, et al. Tofacitinib (CP-690,550) in combination with methotrexate in patients with active rheumatoid arthritis with an inadequate response to tumour necrosis factor inhibitors: a randomised phase 3 trial. The Lancet. 2013, 381:451-460.

3. Fleischmann R, Kremer J, Cush J, et al. Placebo-controlled trial of tofacitinib monotherapy in rheumatoid arthritis. N Engl J Med. 2012, 367:495-507.

4. Fleischmann R, Cutolo M, Genovese MC, et al. Phase IIb dose-ranging study of the oral JAK inhibitor tofacitinib (CP-690,550) or adalimumab monotherapy versus placebo in patients with active rheumatoid arthritis with an inadequate response to disease-modifying antirheumatic drugs. Arthritis Rheum. 2012, 64:617-629.

5. Kremer JM, Bloom BJ, Breedveld FC, et al. The safety and efficacy of a JAK inhibitor in patients with active rheumatoid arthritis: Results of a double-blind, placebo-controlled phase IIa trial of three dosage levels of CP-690,550 versus placebo. Arthritis Rheum. 2009, 60:1895-1905.

6. Kremer JM, Cohen S, Wilkinson BE, et al. A phase IIb dose-ranging study of the oral JAK inhibitor tofacitinib (CP-690,550) versus placebo in combination with background methotrexate in patients with active rheumatoid arthritis and an inadequate response to methotrexate alone. Arthritis Rheum. 2012, 64:970-981.

7. van Vollenhoven RF, Fleischmann R, Cohen S, et al. Tofacitinib or adalimumab versus placebo in rheumatoid arthritis. N Engl J Med. 2012, 367:508-519.

8. Fleischmann RM, Damjanov NS, Kivitz AJ, et al. A randomized, double-blind, placebo-controlled, twelve-week, dose-ranging study of decernotinib, an oral selective JAK-3 inhibitor, as monotherapy in patients with active rheumatoid arthritis. Arthritis Rheumatol. 2015, 67:334-343.

9. Genovese MC, van Vollenhoven RF, Pacheco-Tena C, et al. VX-509 (Decernotinib), an Oral Selective JAK-3 Inhibitor, in Combination With Methotrexate in Patients With Rheumatoid Arthritis. Arthritis Rheumatol. 2016, 68:46-55.

10. Kavanaugh A, Kremer J, Ponce L, et al. Filgotinib (GLPG0634/GS-6034), an oral selective JAK1 inhibitor, is effective as monotherapy in patients with active rheumatoid arthritis: results from a randomised, dose-finding study (DARWIN 2). Ann Rheum Dis. 2017, 76:1009-1019.

11. Genovese MC, Greenwald M, Codding C, et al. Peficitinib, a JAK Inhibitor, in Combination With Limited Conventional Synthetic Disease-Modifying Antirheumatic Drugs in the Treatment of Moderate-to-Severe Rheumatoid Arthritis. Arthritis Rheumatol. 2017, 69:932-942.

12. Takeuchi T, Tanaka Y, Iwasaki M, et al. Efficacy and safety of the oral Janus kinase inhibitor peficitinib (ASP015K) monotherapy in patients with moderate to severe rheumatoid arthritis in Japan: a 12-week, randomised, double-blind, placebo-controlled phase IIb study. Ann Rheum Dis. 2016, 75:1057-1064.

13. Kivitz AJ, Gutierrez-Urena SR, Poiley J, et al. Peficitinib, a JAK Inhibitor, in the Treatment of Moderate-to-Severe Rheumatoid Arthritis in Patients With an Inadequate Response to Methotrexate. Arthritis Rheumatol. 2017, 69:709-719.
