# Supplementary figures and images for: Baricitinib induces LDL-C and HDL-C increases in rheumatoid arthritis: a meta-analysis of randomized controlled trials
Source: Lipids Health Dis. 2019 Feb 18;18:54. doi: 10.1186/s12944-019-0994-7 (PMC6380020; doi:10.1186/s12944-019-0994-7)

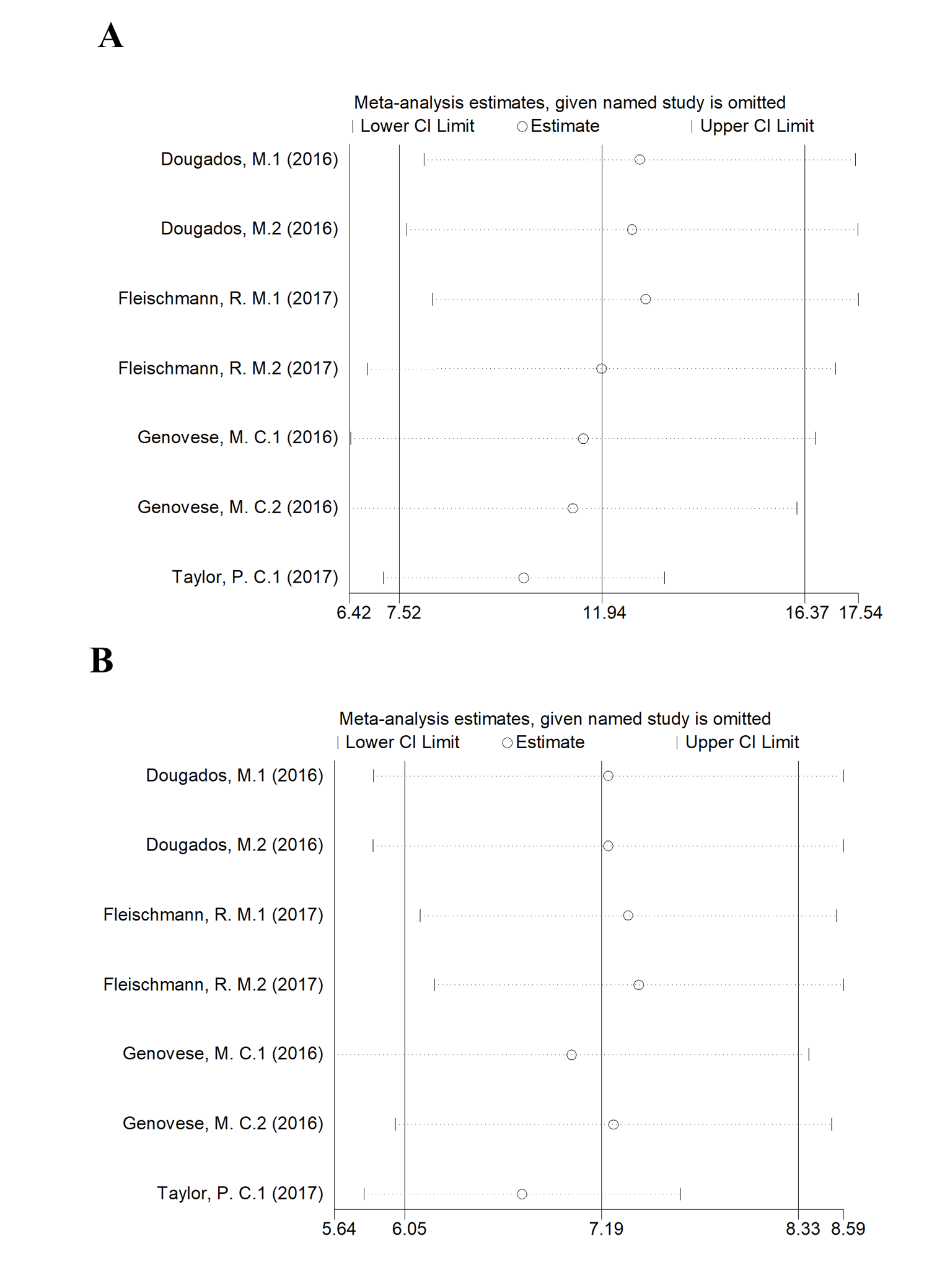

Supplement: Supplementary file 4 — Results of sensitivity analysis. (TIF 361 kb) [file 12944_2019_994_MOESM4_ESM.tif]
