# Supplementary material for: Baricitinib induces LDL-C and HDL-C increases in rheumatoid arthritis: a meta-analysis of randomized controlled trials
Source: Lipids Health Dis. 2019 Feb 18;18:54. doi: 10.1186/s12944-019-0994-7 (PMC6380020; doi:10.1186/s12944-019-0994-7)
Supplement: Supplementary file 5 — Sensitivity analysis of the effect of baricitinib on LDL-C levels (A) and HDL-C levels (B). (DOCX 25 kb) [file 12944_2019_994_MOESM5_ESM.docx]

**Additional file 5.** Sensitivity analysis of net LSM change of LDL-C and HDL-C.

| Study omitted | LDL-C | | HDL-C | |
| --- | --- | --- | --- | --- |
|  | ES (95% CI) | I^2^ | ES (95% CI) | I^2^ |
| Dougados, M.1 (2016)[1] | 12.77 (8.06, 17.48) | 82 | 7.23 (5.87, 8.59) | 53 |
| Dougados, M.2 (2016)[1] | 12.60 (7.68, 17.53) | 85 | 7.23 (5.86, 8.59) | 53 |
| Fleischmann, R. M.1 (2017)[2] | 12.89 (8.25, 17.54) | 84 | 7.34 (6.14, 8.55) | 50 |
| Fleischmann, R. M.2 (2017)[2] | 11.93 (6.83, 17.04) | 87 | 7.40 (6.22, 8.59) | 46 |
| Genovese, M. C.1 (2016)[3] | 11.31 (6.42, 16.19) | 87 | 7.01 (5.64, 8.39) | 55 |
| Genovese, M. C.2 (2016)[3] | 11.53 (6.46, 16.60) | 87 | 7.26 (5.99, 8.52) | 54 |
| Taylor, P. C.1 (2017)[4] * | 10.24 (7.17, 13.31) | 52 | 6.73 (5.81, 7.64) | 0 |

Note, * this study is the major contributor to the heterogeneity of pooled result.

**Reference**

1. Dougados M, van der Heijde D, Chen YC, Greenwald M, Drescher E, Liu J, et al. Baricitinib in patients with inadequate response or intolerance to conventional synthetic DMARDs: results from the RA-BUILD study. Ann Rheum Dis. 2017; 76:88-95.

2. Fleischmann R, Schiff M, van der Heijde D, Ramos-Remus C, Spindler A, Stanislav M, et al. Baricitinib, Methotrexate, or Combination in Patients With Rheumatoid Arthritis and No or Limited Prior Disease-Modifying Antirheumatic Drug Treatment. Arthritis Rheumatol. 2017; 69:506-17.

3. Genovese MC, Kremer J, Zamani O, Ludivico C, Krogulec M, Xie L, et al. Baricitinib in Patients with Refractory Rheumatoid Arthritis. N Engl J Med. 2016; 374:1243-52.

4. Taylor PC, Keystone EC, van der Heijde D, Weinblatt ME, Del Carmen Morales L, Reyes Gonzaga J, et al. Baricitinib versus Placebo or Adalimumab in Rheumatoid Arthritis. N Engl J Med. 2017; 376:652-62.
