# Supplementary material for: Baricitinib induces LDL-C and HDL-C increases in rheumatoid arthritis: a meta-analysis of randomized controlled trials
Source: Lipids Health Dis. 2019 Feb 18;18:54. doi: 10.1186/s12944-019-0994-7 (PMC6380020; doi:10.1186/s12944-019-0994-7)
Supplement: Supplementary file 8 — Risk of bias in the included trials as assessed by the Cochrane risk of bias assessment tool. (DOCX 14 kb) [file 12944_2019_994_MOESM8_ESM.docx]

**Additional file 7. Risk of bias in the included trials as assessed by the Cochrane risk of bias assessment tool.**

|  | **Random sequence generation** | **Allocation concealment** | **Blinding of participants and personnel** | **Blinding of outcome assessment** | **Incomplete outcome data** | **Selective outcome reporting** | **Other bias** |
| --- | --- | --- | --- | --- | --- | --- | --- |
| RA-BEAM | Low risk | Low risk | Low risk | Low risk | Low risk | Low risk | Low risk |
| RA-BUILD | Low risk | Low risk | Low risk | Low risk | Low risk | Low risk | Low risk |
| RA-BEGIN | Low risk | Low risk | Low risk  (double-blind) | Low risk | Low risk | Low risk | Unclear |
| RA-BEACON | Low risk | Low risk | Low risk  (double-blind) | Low risk | Low risk | Low risk | Low risk |
| NCT01469013 | Low risk | Low risk | Low risk  (double-blind) | Low risk | Low risk | Low risk | Unclear |
| NCT01185353 | Low risk | Low risk | Low risk  (double-blind) | Low risk | Low risk | Low risk | Unclear |
| NCT00413660 | Low risk | Low risk | Low risk  (double-blind) | Low risk | Low risk | Low risk | Unclear |
| NCT00550446 | Low risk | Low risk | Low risk  (double-blind) | Low risk | Low risk | Low risk | Unclear |
| NCT00853385 | Low risk | Low risk | Low risk  (double-blind) | Low risk | Low risk | Low risk | Low risk |
| NCT00147498 | Low risk | Unclear | Low risk  (double-blind) | Low risk | Low risk | Low risk | Low risk |
| NCT00814307 | Low risk | Low risk | (Low risk  (double-blind) | Low risk | High risk | Low risk | Low risk |
| NCT00960440 | Low risk | Low risk | Low risk  (double-blind)k | High risk | Low risk | Low risk | Unclear |
| NCT00847613 | Low risk | Low risk | Low risk  (double-blind | Low risk | Low risk | Low risk | Low risk |
| NCT01052194 | Low risk | Low risk | Low risk  (double-blind | High risk | Low risk | Low risk | Unclear |
| NCT2011-004419-22 | Low risk | Low risk | Low risk  (double-blind | High risk | Low risk | Low risk | Low risk |
| NCT01894516 | Low risk | Low risk | Low risk  (double-blind nd) | Low risk | Low risk | Low risk | Unclear |
| NCT01554696 | Low risk | Low risk | Low risk  (double-blind | Low risk | Low risk | Low risk | Unclear |
| NCT01565655 | Low risk | Low risk | Low risk  (double-blind | Low risk | Low risk | Low risk | Unclear |
| NCT01649999 | Low risk | Low risk | Low risk  (double-blind | Low risk | Low risk | Low risk | Unclear |
